# Use of narratives to enhance learning of research ethics in residents and researchers

**DOI:** 10.1186/s12909-015-0329-y

**Published:** 2015-03-10

**Authors:** Kang Sim, Min Yi Sum, Deborah Navedo

**Affiliations:** 1 Department of General Psychiatry, Institute of Mental Health, 10 Buangkok View, 539747 Singapore; 2 Research Division, Institute of Mental Health, 10 Buangkok View, 539747 Singapore; 3 Health Professions Education program, Center for Interprofessional Studies and Innovation , Massachusetts General Hospital Institute of Health Professions, Charlestown Navy Yard, 36 First Avenue, Boston, MA 02129-4557 USA

**Keywords:** Narratives, Research ethics, Engagement, Motivation, Reflective learning

## Abstract

**Background:**

Past didactic pedagogy on biomedical research ethics and informed consent in our program had resulted in passive memorization of information and disengaged learning within psychiatry residents and clinical researchers. The question is how do we better motivate and engage learners within the session. Thus, we incorporated narratives into the learning environment and hypothesised that the use of narratives in the teaching of biomedical research ethics and informed consent would be associated with greater engagement, motivation, understanding, reflective learning and effectiveness of the teaching session.

**Methods:**

The narratives were chosen from the history of research ethics and the humanities literature related to human subject research. Learners were asked to provide post-session feedback through an anonymised questionnaire on their learning session. An outcomes logic model was used for assessment with focus on immediate outcomes such as engagement, motivation, understanding and reflective learning.

**Results:**

Overall, 70.5% (N = 273) of the learners responded to the questionnaire. Amongst the respondents, 92.6% (N = 253) of the participants ranked use of narratives as most helpful in appreciating the historical context of research ethics and informed consent in research. The majority felt engaged (89.8%, N = 245), more motivated to learn (77.5%, N = 212) and better equipped (86.4%, N = 236) about the subject matter. Better appreciation of the learning topic, engagement, motivation to learn, equipping were strongly correlated with the promotion of reflective learning, effectiveness of teaching, promotion of critical thinking and overall positive rating of the teaching session on research ethics (all p < 0.001). Multivariate analyses found that the use of narratives was associated with higher overall rating of the teaching session (p = 0.003) and promotion of critical thinking (p = 0.02).

**Conclusion:**

Results revealed that the use of narratives could enhance engagement, appreciation of biomedical research ethics and informed consent, and address underlying motivational factors behind learning and understanding of research ethics.

## Background

Biomedical research ethics is an integral part of the research training programs for our psychiatry residents, clinical researchers and research staff including clinical research co-ordinators and professionals. Training programs such as the “National Psychiatry Residency Research Training Module” and the “Good Clinical Practice Guidelines for Clinical Research Course” which are conducted within the National Healthcare Group Offices, Singapore, seek to provide better learning of the ethical, regulatory, procedural, legal, safety and quality assurance aspects in biomedical research locally in terms of better understanding, reflection and application of principles of ethics in practice. The Research Training Module is part of the curriculum for our National Psychiatry Residency Program. Within the local context, as part of IRB requirements, all clinical researchers involved in clinical trials need to undergo the Singapore Guidelines on Good Clinical Practice (SGGCP) course to familiarize themselves with the ethical principles related to medical research.

There are several learning needs of the learners for such a research module. There is a need to be able to understand the rationale for current research ethics guidelines, appreciate the relationship of informed consent to broader ethical guidelines such as the Belmont report, Declaration of Helsinki, and to articulate and consider the elements of informed consent within research protocols. However, observations by the teaching faculty found that past didactic pedagogy on specific topics such as “Research Ethics and Informed Consent” had resulted in passive, disengaged and unmotivated learners within a dry knowledge context, which in turn led to ineffective application of ethical principles to practice. Informal conversations with these learners had revealed the consistent theme that past learners were not able to see the relevance and congruence of ethical principles to their own clinical contexts, background and past experience in research.

The goals of these teaching sessions on Research Ethics and Informed Consent are: (1) to better engage and motivate participants in the learning about research ethics such that there is better understanding, reflection, and application of research ethical principles, (2) to understand the relevance of this historical background to clinical and research setting of the residents, researchers and research staff, and (3) to reflect on the components and zeitgeist of informed consent process which is more than just a form.

### Theories of learning

The learners come from diverse backgrounds varying from psychiatry residents, researchers who are mainly clinicians, and research staff including research office staff, and staff from clinical research organisations, and thus they bring to the session varied levels of prior research experience. However, they would all be considered adult learners with relevant learning preferences and needs. As adult learners [1], they need to know the rationale of learning what they are learning, in this instance, research ethics and informed consent (question of why it is relevant). They should be able to activate their prior knowledge in terms of linking the topic to their past and present experiences of having been involved in research protocols and recruitment of subjects in research studies and clinical trials (question of how is it relevant). The adult learners are imbued with a sense of readiness to learn and entrusted with self-directed learning. In the context of understanding the why and how of relevance, there is then a natural motivation to pursue and to learn more about the learning topic (acquisition of what is relevant).

The constructivist theory of learning [2] has parallels with adult learning principles outlined above in that both theories of learning are concerned about the context of learning and the learner’s readiness to learn. Constructivism focuses on the learner’s experiences and allows for the information to be reflected upon, processed, organized and constructed into new ideas or concepts in the process of learning. In terms of motivation to learn, the learners need to be able to see the discrepancy or gap between the relevance and amount of history behind the current ethical frameworks, regulatory and quality assurance processes and determine their own level of understanding at this juncture [3]. Motivation to learn is then a function of the drive to attend to the individual’s perceived need in the relevant context of the research module. This would then lead the learner to make decisions about engagement in specific learning activity which is related to the expectation of enhanced understanding of biomedical research (expectancy), ability of activity to improve functioning as a biomedical researcher (instrumentality), and overall personal satisfaction (valence) [4]. In this context, narratives potentially allow the adult learner to understand, apply, and evaluate the relevance (why, how, what) of the case stories told, and to further create the meanings of these narratives for themselves.

### Use of narratives in teaching research ethics

Whilst narratives can be considered as a phenomenon or method, our focus is on the former in this study. People by nature lead storied lives and tell and retell their stories as they reflect and explain themselves to others including their dilemmas [5]. In contrast to logical scientific knowledge which attempts to clarify universal truths by transcending the individual, narrative knowledge seeks to illuminate universal truths by understanding the individual [6]. The focus of narrative is on the personal human experience, is wholistic and has an important place in literature and social sciences [5]. Although narratives have been increasingly used in medical education, there is sparse literature on its specific use in the teaching of biomedical research ethics [7].

Using narratives in teaching biomedical ethics is pertinent and significant based on several considerations. Narratives tell of how people change over time including character and moral development. This allows us to enter the lived experiences of the characters within narratives and understand and share in their dilemmas and struggles, which fits well with ethical considerations in real life within the biomedical context [7]. How someone listens to the narratives and provides alternative narratives can allow a mental frame to develop for contemplation of a wider or smaller range of options for different ethical issues. In one sense, narratives are stories which organize and evaluate the world hence their appropriate use can lend contextual relevance and teach us helpful principles from the history of research ethics including issues such as vulnerable populations, respect for the person [8]. In addition, they teach us what to look for and what can be ignored, what to value and hold in contempt [9], hence they can give ethical insight and wisdom through case histories regarding ethical decision making and the factors to be taken into consideration. Narratives reflect culture and their value is in giving clarity without betraying the complexity of life in flux [10]. This can give balance in the context of biomedical research ethics where there are often inter-related and interactive elements to be considered in the context of the research population and humanistic factors, protocol, funding body, researchers and regulatory body.

Importantly, they give human voice to what is said [11] and brings us back to the personal and inter-personal aspects of biomedical research and not lose the “person” in the process of scientific scrutiny and analyses. They also allow the learner to make sense of the story as they create and construct meaning on their own thus making static bioethical principles come alive [7].

### Aim and hypothesis

For these reasons, narratives were incorporated into the learning environment and teaching topic on “Research ethics and informed consent”. In light of the scant literature on the use of narratives in teaching of biomedical research ethics, we sought to investigate whether the use of narratives could enhance engagement and motivation to learn research ethics in this study. Based on these potential benefits of using narratives in research ethics teaching, we hypothesised that the use of narratives in the teaching of biomedical research ethics and informed consent would be associated with greater engagement, motivation, understanding of topic, reflective learning and perceived effectiveness of the teaching session.

## Methods

An update of the research training module was planned and implemented for all of the learning groups using the narrative approach. The efficacy was evaluated by surveying the participants with focus on immediate outcomes following the teaching session.

### Teaching session

The teaching session on Research Ethics and Informed Consent was part of the research training module within the “National Psychiatry Residency Training Program” and the “Good Clinical Practice Guidelines” course organised by the National Healthcare Group Cluster Offices. The psychiatry residents within the former course were all in their second year of training and had exposure to research projects either in earlier or current postings. The participants in the latter course were researchers and staff from the research office or clinical research offices and who were actively involved in research projects or clinical trials in their course of work.

### Use of narratives

The outcomes logics model [12] was adapted for our study, and included inputs, activities, outputs, outcomes and impact (see Table 1). Four narratives that were incorporated into the teaching session were garnered from the history of research ethics related to the Nuremberg Code (Nazi experiments conducted during the Second World War), Belmont Commission report (Tuskegee syphilis project) and from the literature related to human subject research (stories of Henrietta Lacks and Jesse Gelsinger). These stories were incorporated and interspersed within the teaching sessions lasting about 60 minutes and prominent themes relevant to breaches of ethical principles can be raised such as respect for autonomy, beneficence, non-maleficence, and distributive justice. During the ensuing facilitated interactive discussion about the stories shared, the whole learner group varying from 10 to 40 individuals was encouraged to reflect about the ethical issues of concern, breaches and observations regarding issues such as informed consent.

**Table 1:** **The Outcomes Logic Model for the teaching session for psychiatry residents and research staff**

Inputs	Outputs		Outcomes -- Impact	
	*Activities*	*Participation*	*Immediate*	*Intermediate*
--Funding from NHG Headquarters/Education and Research Development Office	--Use of narratives	--Overall number of learners from psychiatry residency program and research offices	--Better engagement and motivation to learn compared to before the teaching session	--Greater involvement in research projects
--Faculty and staff time	---Support systems such as administrative support to publicise and organize the session, and management of logistics		--Better engagement and motivation to learn compared to before the teaching session	--Practical experience of submission of protocols to IRB with proper informed consent description and documentation
--Facilities within learning environment	---Facilitated group discussion		--Increased understanding of the background and principles of biomedical research ethics and informed consent	--Continuous medical education to appreciate history of research ethics to inform current research practice of informed consent
	---Availability of tutor for clarification of concepts		--Greater reflective learning	
			--Overall better equipping of the principles of biomedical research ethics	

### Anonymised questionnaire

An anonymised questionnaire, comprising of rated items and open ended questions, was designed and administered at the end of the teaching session and filling of the questionnaire by the learners was completely voluntary, hence, written informed consent was not obtained from participants to ensure their anonymity.

The majority of the relevant items in the questionnaire were rated along a 5 point Likert Scale from Strongly Disagree (1 point), Disagree (2 points), Neutral (3 points) to Agree (4 points) and Strongly Agree (5 points). The questions included items such as: (1) whether the narratives helped in appreciation and understanding of the topic, (2) whether learners felt more engaged, motivated and better equipped about the biomedical research principles after the teaching session when compared to before the teaching session, (3) promotion of reflective learning, critical thinking, and (4) effectiveness of teaching. The overall rating of teaching session on research ethics and informed consent was rated along a 6 point Likert scale from poor (1-2 points) to average (3-4 points) and excellent (5-6 points). The learners were also asked open ended questions about specific aspects of the teaching session that helped their learning as well as what they desired to see more being done to help with their learning.

### Ethical approval

This study was approved by the cluster Institutional Review Board, National Healthcare Group Domain Specific Review Board (NHG DSRB) as an exempt study (NHG DSRB Ref: 2014/00422).

## Results

Overall, 273 out of 387 (70.5%) learners responded to the questionnaire. Amongst the respondents (17 psychiatry residents and 256 researchers and research staff), 92.6% (N = 253) of the participants agreed or strongly agreed that the use of narratives was most helpful in appreciating research ethics and informed consent in research. The majority agreed or strongly agreed to feeling engaged (89.8%, N = 245), more motivated to learn (77.5%, N = 212), and better equipped (86.4%, N = 236) about the subject matter.

Specifically, better appreciation of the learning topic, engagement, motivation to learn, equipping were strongly and respectively correlated with the promotion of reflective learning, effectiveness of teaching, promotion of critical thinking and overall positive rating of teaching session on research ethics (all p < 0.001) based on Pearson’s correlation analyses. Compared with researchers and research staff, psychiatry residents felt that teaching with narratives was more effective (p = 0.033) and promoted better reflective learning (p = 0.001). Multivariate linear regression analyses found that the use of narratives was associated with higher overall rating of the teaching session (p = 0.003) and promotion of critical thinking (p = 0.02).

The qualitative feedback based on open coding for the open ended questions further supported the quantitative findings in that the majority of learners (92.8%, N = 254) shared that the use of narratives had aided their learning. The rest (7.2%, N = 19) listed other factors that helped in their learning including the style of delivery, humour, facilitation of discussion, stepping off podium and speaking to participants, emphasis on main points and sharing of personal experience from a fellow researcher.

In addition, 43 participants gave comments about areas for improvement to aid learning and the main area mentioned was longer session with more stories (28 out of 43 learners, 65.1%). The other areas of improvement included more hands-on-practice on drafting an informed consent document, live quizzes to test knowledge, and asking more questions during the session.

## Discussion

To the best of our knowledge, this is the first study which examined the use of narratives in teaching research ethics and their impact on learning. There were several main findings in this study. First, the majority of learners found that the use of narratives was helpful in learning the topic of informed consent in the context of research ethics. Second, the learners also reported feeling more engaged, motivated and equipped compared to before the session. Third, greater engagement and motivation seemed to relate to greater reflective learning and better overall learning, especially in the small group of psychiatry residents.

Narratives were found to be helpful in enhancing appreciation of the teaching topic in this study and various reasons are plausible. Stories are essential as people tell stories to live and make sense of their experience [13]. Our findings of better appreciation of the learning topic and reflection were consistent with the constructivist theory of learning which allows for consideration of how the atrocities committed during the Nazi experimentations had led to immeasurable pain and suffering for the victims and families. These reflections further allow for some meaning to be constructed with subsequent awareness about the measures that were taken in order to protect vulnerable populations in research. In the narrative of the Tuskegee syphilis case study and consistent with constructivism, our findings of better appreciation and overall learning reminded the learner about the need to reflect upon the issue of respect for the person and human subject in the observational cohort study as well as the vulnerability of special populations thus requiring better care and explanation of the details and implications of the study [9]. Furthermore, narratives can give clarity about the relevant biomedical issues of concern without oversimplifying the nature of the circumstances or complexity of daily lives in the different societies for the learner [14,15]. The story of Henrietta Lacks must be read and heard in the context of the research scene in 1930s when consent may not be so widely discussed and the future use of any biopsied biological tissues may be taken for granted then. The severity of her cervical cancer, concerns about her family, treatment regimens and prognosis and other issues could be further explored and meaning derived from these discussions.

We found that these narratives better engaged and motivated the learners in their process of learning. This may be explained using needs reduction theory in pointing out the gap in the knowledge and experience of the learner between what they ought to know and what they know [3]. In this aspect, the narratives can highlight to the learner the background to the development of the various codes of research conduct or reports such as the Nuremberg Code, Declaration of Helsinki, Belmont report and set a yardstick to be measured against their understanding of the history of biomedical research ethics. The discrepancy between what ought to be known and what is known can spur the learner towards knowing and appreciating more about research ethics. In terms of engagement, there are several learner factors influencing the decision to pursue the topic of interest which were related to the expectation of enhanced understanding of biomedical research (expectancy), ability of activity to improve functioning as a biomedical researcher (instrumentality), and overall personal satisfaction (valence) in the learner [4].

In addition, we found that the use of narratives encouraged reflective learning and critical thinking in the learners within the session. This is congruent with earlier medical literature which expounded on the ability of narratives to increase the empathy, trust, reflection as well as professionalism of the learners [6]. This may be used in reflective writing which involves processes such as interpretation, reasoning, comparisons and contrasts. It has been thought that there should be narrative humility and an avoidance of summarizing and making moral judgements at the end of the story as this may affect the wholistic nature of narratives via oversimplification, overgeneralization or judgementalism [16].

**Figure 1:**
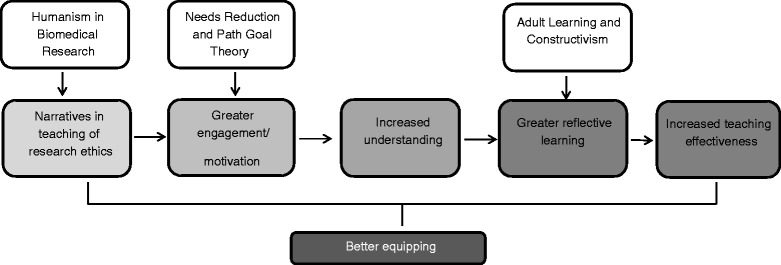
**How narratives help in the teaching of biomedical research ethics.**

Overall, how do narratives aid the learning and teaching of research ethics? We present one possible pathway which is supported by our findings and anchored by different learning theories (refer Figure 1). Medicine and biomedical research involves people (including patients, healthy controls, caregivers, therapists, researchers) and hence they are intimately related to the storytelling enterprise. The use of narratives evokes the humanism in the learner and their relationships in the learning of medicine and biomedical research. The discrepancy highlighted by the use of narratives between what ought to be known and what is known in the learner stimulates interest in the teaching topic [3]. The perceived ability to fill the gap in knowledge and fulfill the desirable goals of instrumentality (performance), valence (personal satisfaction), and expectancy (perception of teaching filling the gap) enhances the engagement in the learning process [4]. The better motivation and engagement facilitates understanding and reflective learning as the content of the narratives were considered, compared, contrasted, and interpreted with convergence of meaning.

We found that the use of narratives was especially helpful for the psychiatry residents in terms of promoting reflective learning and teaching effectiveness which can be consistent with earlier work in this area. Narratives allow the learner to attend to the human voice behind what is told and allows the person behind the bioethical principles to appear which aligns with psychiatry training focussing on first listening to the patient [11]. In the clinical context, narratives rehumanises clinician investigator-patient relationships in the context of research which psychiatry residents could identify with during their residency training [17]. Charon had proposed that the use of narratives promotes narrative competence as it promotes the ability to acknowledge, absorb, interpret and act on the stories, and thus engendering empathy, trust, reflection and professionalism in the learners [6].

There are limitations to the use of narratives in the teaching of research ethics based on earlier readings. Stories can be indeterminate and narratives in research ethics cannot provide formulaic prescriptions and provide clear procedures to aid decision making in all cases of research [18]. For example, the issues highlighted by the Tuskegee syphilis study about respect for autonomy and vulnerable populations need to be adapted in our understanding of genetic research using genome wide association tests in the era of personalized medicine. In the context of such potential altruistic databases, issue about confidentiality of data may not necessarily be neglected. In addition, narratives may conceal as much as they reveal about the characters in the stories [19]. There is thus a need for accountability and to be as factual as possible in terms of preparing the narrative for teaching research ethics. Narratives can invoke pain and suffering as much as alleviate fears, secrecy, loss and death. There may be concerns about re-victimisation of the characters involved and hence there is a need to protect confidentiality of persons involved in the stories and to get permission if necessary. There may be learners who do not prefer learning via narratives and prefer facts and figures to sharing of stories [20].

Future work will want to look at refining the appropriate use of narratives and type of narratives for different learning needs within research and clinical settings. Developing a practical framework can also increase learning satisfaction. One model of such narrative inquiry in the context of research ethics could follow the framework of Charon [21]. The framework includes following a narrative thread, tolerating ambiguity, adoption of multiple and contradictory points of view, entering narrative’s reality and understanding how reality is making sense, seeing the use of image and metaphor and increasing imagination and listening skills.

## Conclusion

In conclusion, we found that the use of narratives can enhance the appreciation of biomedical research ethics and informed consent, engagement of learners, and address underlying motivational factors behind learning. Medicine including biomedical research is about people and patients, thus it is a story telling enterprise [22]. Narratives can enliven bioethical principles, allows insight, shape bioethical principles, and can be useful for teaching proper attitudes and values in the conduct of biomedical research.
